# Comparison of Epidermal Barrier Integrity in Adults with Classic Atopic Dermatitis, Atopic Prurigo and Non-Atopic Prurigo Nodularis

**DOI:** 10.3390/biology10101008

**Published:** 2021-10-07

**Authors:** Regina Fölster-Holst, Rahel Reimer, Claudia Neumann, Erhardt Proksch, Elke Rodriguez, Stephan Weidinger, Mohamad Goldust, Eckhard Hanisch, Stephan Dähnhardt-Pfeiffer, Sandra Freitag-Wolf

**Affiliations:** 1 Department of Dermatology, Venereology and Allergology, University Hospital Schleswig-Holstein, Campus Kiel, 24105 Kiel, Germany; cneumann@dermatology.uni-kiel.de (C.N.); eproksch@dermatology.uni-kiel.de (E.P.); erodriguez@dermatology.uni-kiel.de (E.R.); sweidinger@dermatology.uni-kiel.de (S.W.); 2 Department of Dermatology, University Medical Center Mainz, 55101 Mainz, Germany; mgoldust@uni-mainz.de; 3 Neubourg Skin Care GmbH & Co. KG, 48268 Greven, Germany; hanisch@neubourg.com; 4 Microscopy Services Dähnhardt GmbH, 24220 Flintbek, Germany; 5 Institute of Medical Informatics and Statistics, University Hospital Schleswig-Holstein, Campus Kiel, 24105 Kiel, Germany

**Keywords:** atopic dermatitis, prurigo nodularis, epidermal barrier

## Abstract

**Simple Summary:**

Atopic dermatitis, also called neurodermatitis, is one of the best-known chronic inflammatory skin diseases with eczema, strong itch and dry skin. It is based on an impaired skin barrier with changes in the fats and proteins of the horny layer, which leads to a disturbed water balance. Prurigo nodularis is a less common and intractable chronic skin disease with very itchy nodules. It can be associated with atopic dermatitis (so called atopic prurigo) or with many other diseases. The aim of this study was to compare these three skin diseases classic atopic dermatitis, atopic prurigo and non-atopic prurigo nodularis with healthy control subjects with regard to their skin barrier. In all three disease groups, we then found marked disease severity, reduced water content and increased water loss through the skin, thickening and inflammation of the skin, altered levels of certain proteins and reduced fat layers, all of which demonstrate a severe disturbance of the skin barrier. The main therapeutic consequence is that basic barrier repair, as established for patients with atopic dermatitis in the form of consistent skincare, could also be crucial for patients with non-atopic prurigo nodularis due to this verified skin barrier damage.

**Abstract:**

A deficient epidermal barrier is a key feature of atopic dermatitis (AD) and comprises altered lipid and protein content and composition of the stratum corneum resulting in disturbed water balance. Clinically, eczematous lesions on dry skin and pruritus develop. Pruritic nodules occur in prurigo nodularis (PN), another chronic skin disease, which can be associated with atopy. We aimed at comparing the three clinical pictures, classic AD, atopic prurigo (AP), and non-atopic PN, to healthy controls regarding the epidermal barrier. We determined clinical parameters and performed biophysical measurements, histology/immunohistochemistry, electron microscopy, and molecular biological analysis. We found distinctively elevated clinical scores, reduced hydration and increased transepidermal water loss, epidermal hyperplasia and inflammation reduced filaggrin and increased loricrin and involucrin expression, as well as reduced intercellular lipid lamellae in all three disease groups. These findings show a severe disruption in epidermal barrier structure and function in all three disorders so that epidermal barrier impairment is now proven not only for AD but also for PN.

## 1. Introduction

Atopic dermatitis (AD) is one of the most common chronic inflammatory skin diseases that typically occurs in early childhood and can relapse or persist in adulthood; an adult-onset form exists as well. The characteristic clinical features of AD are various eczematous skin lesions with chronic lichenification, accompanied by intense itch and often very dry skin. AD is commonly associated with a personal and/or family history of atopy. Its pathophysiology is based on three main traits, which are influenced by genetic and environmental risk factors: a dysfunctional epidermal barrier, a dysbiosis of the skin microbiome, and aberrant type-2 dominated immune responses mainly driven by antigen-specific T cells, all three of which show complex interactions and drive cutaneous inflammation [1,2,3,4,5].

An abnormal lipid composition, such as a decrease in certain ceramides, which in turn evokes both a reduction as well as an impaired organization of the intercellular lipid lamellae (ICLL) of the stratum corneum (SC), and a disturbance of the protein expression, such as a filaggrin deficiency in the cornified envelope (CE) of the SC, as well as other structural changes in the SC, collectively lead to a disruption of the epidermal barrier integrity. This results in a dysfunction of the epidermal barrier permeability, such as an increase in transepidermal water loss (TEWL) and a reduction in water content of the SC both in lesional and non-lesional skin [4,5,6,7,8,9]. Gene variants, especially in the *filaggrin* (*FLG*)-gene, contribute to this barrier impairment and increase the risk of developing severe AD [10,11,12].

A variant of AD is atopic prurigo (AP, prurigo-type of AD), characterized by erythematous, excoriated papules or nodules as well as intense pruritus and particularly affects adult patients with longstanding AD or late-onset AD [1,2,3]. A comparable clinical picture is seen in prurigo nodularis (PN), which was recently defined as a subtype of chronic prurigo, a chronic skin disease of low prevalence [13,14,15,16]. PN is most frequently associated with AD as atopic prurigo but may also occur in non-atopic patients in many other pathologies, other dermatoses, and systemic disorders, e.g., renal failure or liver diseases, malignancies, endocrinopathies or infections, neurological or psychological diseases [14,17,18]. The exact pathomechanisms underlying PN are not yet fully understood; they include neurogenic inflammation and neuronal dysregulation, which are thought to be driven by eosinophils, mast cells, and T cells and triggered by repeated scratching [19].

Since barrier dysfunction of the entire skin is a key molecular feature of AD, the so-called basic therapy with continuous and liberal use of emollients and moisturizers is an important part of disease management [1,20]. Emollients are also recommended in PN to prevent xerosis that triggers or exacerbates itch due to general barrier damage [13,21,22]. Topical corticosteroids and calcineurin inhibitors have an anti-inflammatory effect in AD and PN, in addition to calcipotriol and capsaicin in PN. However, all these topical drugs are insufficient in severe forms. Systemic immunosuppressants such as cyclosporine A and antihistamines are also used in both conditions, antidepressants and gabapentinoids are often used in PN as well. In PN, the inhibitors of neurokinin-1- and opioid-receptors have been shown to improve the disease. Dupilumab, a human monoclonal antibody that blocks the common receptor component for interleukin (IL)-4 and IL-13, is currently approved for AD but is also an effective drug in PN. The clinical response to dupilumab has a later onset in PN compared to AD, interestingly especially in patients with AP [3,14,20,21,23,24,25].

The aim of the current study was to compare the three presented chronic, pruritus-associated skin diseases, classic AD, AP, and non-atopic PN, to skin healthy adults regarding the structure and function of the epidermal barrier, which is very well studied in AD, but hardly at all in PN, in order to be able to derive consequences for diagnostics or therapy on the basis of possible differences or similarities.

To examine the epidermal barrier, several clinical parameters were used for analysis: comprehensive clinical history, a scoring system for atopic dermatitis disease severity (i.e., Severity Scoring of Atopic Dermatitis [SCORAD]) and a quality of life assessment (i.e., Dermatology Life Quality Index [DLQI]). In addition to these, TEWL and skin hydration measurements were performed as biophysical parameters, while the expression of three important epidermal differentiation proteins as immunohistological parameters and four common *FLG*-mutations as molecular genetic parameters were analyzed. For direct evaluation of the barrier condition electron microscopy was performed, which is able to visualize the lipid lamellar organization between the corneocytes of the SC.

## 2. Materials and Methods

### 2.1. Subjects and Study Design

The study was carried out at the Department of Dermatology, University of Kiel, Germany, between October 2015 and June 2016. The investigations were conducted on 35 adults 18–60 years of age: 9 patients with AD, 7 patients with AP, 9 patients with PN, and 10 healthy controls (24 female and 11 male). All patients were recruited by the Department of Dermatology of the University Hospital in Kiel, Germany, and had to demonstrate a dermatologist-confirmed diagnosis of the respective disease—in the atopic groups according to the criteria of Hanifin and Rajka [26]—as well as skin lesions visible at the time of examination. The examinations were performed on lesional and non-lesional skin directly side by side. An attempt was made to choose the same localization, i.e., the lower extremity, for all measurements and samples.

### 2.2. Medical History and Clinic

The subjects were interviewed using questionnaires about their medical history and then examined clinically to evaluate several criteria: the severity of disease using the SCORAD [27], the level of itch intensity using the Numerical Rating Scale (NRS) for pruritus [28], and the patient’s dermatological quality of life using the DLQI [29]. In addition, common and associated features such as xerosis were documented with severity levels from 0 (=not present) to 3 (=strongly pronounced).

### 2.3. Biophysical Measurements

To assess the permeability barrier function, the measurements of the biophysical parameters, TEWL and SC hydration, were performed using the Corneometer^®^ CM 825 and the Tewameter^®^ TM 300 (Courage + Khazaka Electronic GmbH, Köln, Germany) by standardized methods as described previously by Jensen et al. [30].

### 2.4. Histology/Immunohistochemistry

To investigate the expression of the CE-proteins filaggrin, loricrin, and involucrin, 4 mm punch biopsies were taken: Two to each patient (one lesional and one non-lesional) and one to each control. Hematoxylin and eosin (HE) staining served for the evaluation of epidermal thickness, inflammation, hyperkeratosis, and hypergranulosis. The preparation for immunohistochemistry according to the (Strept)Avidin-Biotin Complex (ABC) method and the antibodies used have been described previously by Jensen et al. [29]. The histologist was blinded, five random fields of view were examined in each histological section. The sections were evaluated descriptively according to semi-quantitative aspects using a score from grade 0 (=normal) to grade 3 (=strong).

### 2.5. Electron Microscopy

The non-invasive SC sampling using Lipbarvis^®^, their processing, and the following analysis of the ICLL of the SC using transmission electron microscopy (TEM) was carried out as described in previous studies by Dähnhardt-Pfeiffer et al. [6]. In this technique, morphometric analysis of the length of the ICLL in the intercellular space (ICS) in relation to the surface area of the ICS is calculated with respect to a reference value of 1000 nm^2^ (normalized ICLL [nICLL]). This ratio allows a semi-quantitative statement about the skin barrier integrity [31].

### 2.6. FLG-Mutations

*FLG* variants R501X, 2282del4, R2447X, and S3247X were analyzed with the TaqMan allelic discrimination method (Applied Biosystems, Foster City, CA, USA) after DNA preparation from blood samples. TaqMan SNP Genotyping was carried out with 3.125 ng of dried genomic DNA in 384-well genotyping reactions and 5 µL reaction volumes following standard procedures based on Applied Biosystems reagents. Probe detection was performed with the Applied Biosystems 7900HT Fast Real-Time PCR system [32].

### 2.7. Statistics

The results were statistically analyzed using SPSS (version 25) and the R (version 3.6) software. Descriptive statistics for qualitative and quantitative values are given as relative frequencies or means ± standard deviation (SD) or medians with first and third quartile. Differences between two given groups were determined by using the χ^2^ test (or Fisher’s exact test) for nominal data, the Mann-Whitney U test for ordinal and metric, not normally distributed data, and the two-sample t-test (or Welch’s test) for metric, normally distributed data. For the overall analysis of more than two groups, the ANOVA or Kruskal-Wallis test were used, as appropriate. Moreover, the analysis was revised by regression models in order to identify potential confounders such as age, gender, or measurement localization, which yielded similar results. All statistical tests were performed two-sided for unrelated samples, not adjusted for multiple testing because of the explorative manner of the study. The local level of significance was set to 5%.

## 3. Results

The numerical values for all parameters are listed in Table 1.

### 3.1. Medical History Features

The age at disease onset was significantly higher in the PN-group than in the AD- (*p* = 0.006) and AP-group (*p* = 0.029). The AP-group suffered significantly more often from allergic asthma than the PN-group (*p* = 0.005). The AD-group had significantly more frequently pollen allergy than all the other three groups AP (*p* = 0.041), PN (*p* = 0.002) and C (*p* = 0.001). The AP-group offered significantly more often a positive family history of atopic diseases within first-degree relatives than the PN-group (*p* = 0.041); the AD-group also showed this positive family history clearly, although not significantly.

In addition, the AP- and PN-groups had significantly higher BMI values in the overweight range than the AD-group (*p* = 0.005, *p* = 0.003) and the C-group (*p* = 0.001, *p* < 0.001).

Only the AD-group used daily skincare significantly more often than the C-group (*p* = 0.011). There were no significant differences between the disease groups.

### 3.2. Clinical Characteristics

**Figure 1:**
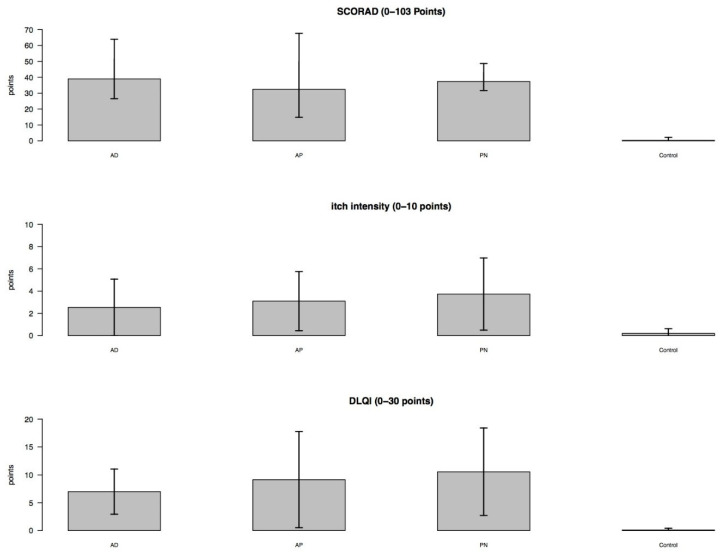
Clinical parameters (bar plots with standard deviation). SCORAD: severity scoring of atopic dermatitis; DLQI: dermatology life quality index; AD: classic atopic dermatitis; AP: atopic prurigo; PN: non-atopic prurigo nodularis.

The patients in the different disease groups AD, AP and PN presented pronounced clinical pictures, which were reflected in the collected clinical parameters: Compared to the C-group, significantly increased SCORAD values (each *p* < 0.001), current itch intensity levels (*p* = 0.002, *p* = 0.006, *p* = 0.014) and DLQI values (*p* < 0.001, *p* = 0.002, *p* < 0.001) were observed. There were no significant differences between the disease groups (Figure 1). 

In addition, xerosis was significantly increased in the AD group compared to the AP (*p* = 0.031), PN (*p* = 0.045) and C (*p* = 0.003) groups.

### 3.3. Hydration and TEWL

**Figure 2:**
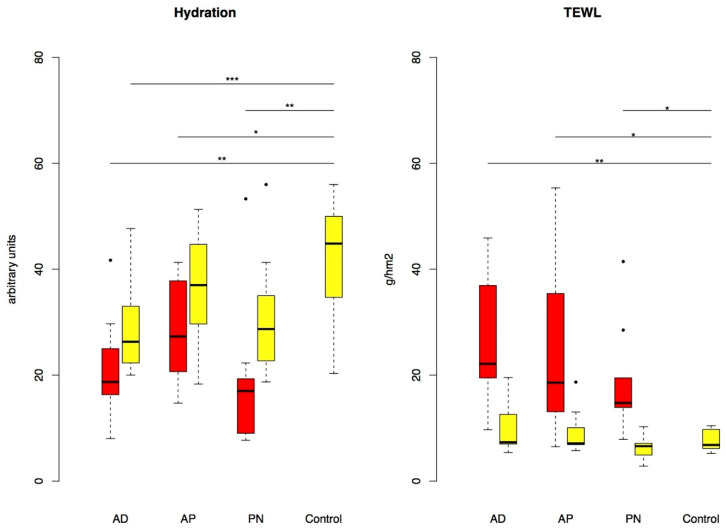
Hydration and TEWL in lesional (red) and non-lesional (yellow) skin (boxplots with outliers given as single dots). *p*-value: * *p* < 0.05, ** *p* < 0.01, *** *p* < 0.001. TEWL: transepidermal water loss; AD: classic atopic dermatitis; AP: atopic prurigo; PN: non-atopic prurigo nodularis.

In all three patient groups AD, AP and PN, there were significant reductions in the SC hydration (*p* = 0.001, *p* = 0.026, *p* = 0.001) and significant increases in TEWL (*p* = 0.001, *p* = 0.04, *p* = 0.015) in lesional skin compared to the C-group. If we focus only on non-lesional skin, there was a significant reduction in SC hydration only in patients with AD (*p* = 0.012). There was no significant difference comparing the patient groups (Figure 2).

### 3.4. Histological Features and Epidermal Differentiation

Histology revealed a significantly thickened epidermis and a significant inflammatory infiltrate in lesional skin of all three disease groups AD (each *p* < 0.001), AP (each *p* = 0.002), and PN (each *p* < 0.001) compared with the C-group. The disease groups showed no significant differences among each other. 

A significant hyperkeratosis and a significant hypergranulosis were detected in lesional skin of all three disease groups AD (each *p* < 0.001), AP (each *p* = 0.001), and PN (each *p* < 0.001) compared with the C-group. Additionally, in the AD-group, the hyperkeratosis in non-lesional skin was significantly higher than in the C-group (*p* = 0.041) and even higher than in the prurigo groups AP (*p* = 0.001) and PN (*p* < 0.001). 

In all three disease groups, immunohistochemistry showed a reduction in filaggrin and a distinct increase in loricrin and involucrin expression.

**Figure 3:**
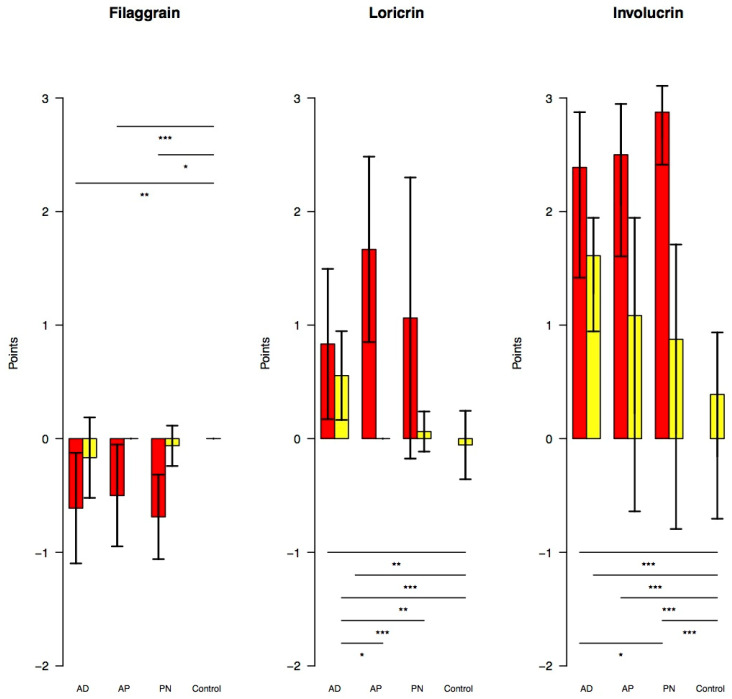
Expression of filaggrin, loricrin and involucrin in lesional (red) and non-lesional (yellow) skin (bar plots with standard deviation). *p*-value: * *p* < 0.05, ** *p* < 0.01, *** *p* < 0.001. AD: classic atopic dermatitis; AP: atopic prurigo; PN: non-atopic prurigo nodularis.

**Figure 4:**
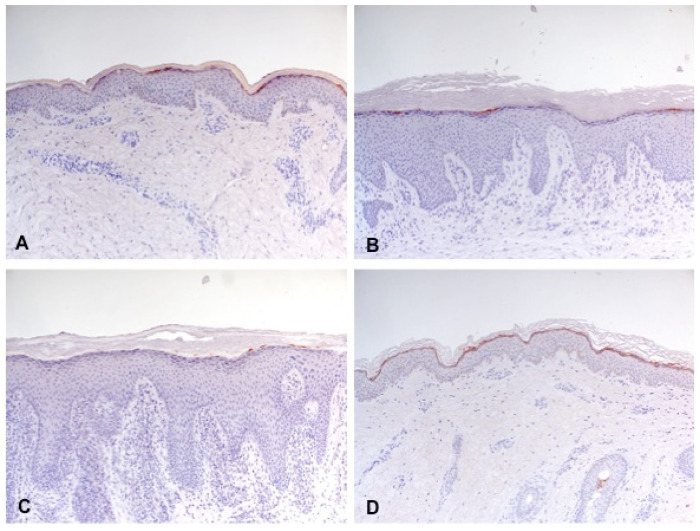
Reduced expression of filaggrin (partially interrupted brown staining line) in lesional skin of classic atopic dermatitis (**A**), atopic prurigo (**B**), and non-atopic prurigo nodularis (**C**) compared with healthy controls (**D**) shown by immunohistochemistry (100-fold magnification).

Filaggrin was only significantly reduced in lesional skin of all three disease groups AD (*p* = 0.002), AP (*p* = 0.011), and PN (*p* < 0.001) compared with the C-group. The disease groups showed no significant differences among each other (Figure 3 and Figure 4). 

**Figure 5:**
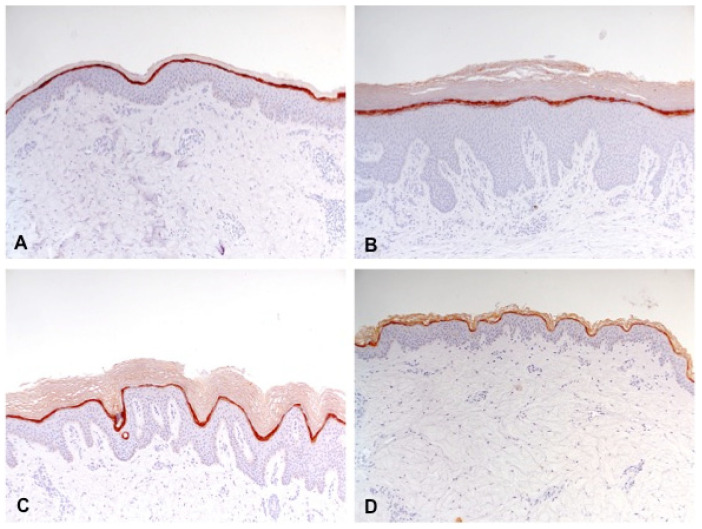
Increased expression of loricrin (thickening and staining density of the brown staining line) in lesional skin of classic atopic dermatitis (**A**), atopic prurigo (**B**), and non-atopic prurigo nodularis (**C**) compared with healthy controls (**D**) shown by immunohistochemistry (100-fold magnification).

Loricrin was significantly increased compared with the C-group in lesional and non-lesional skin of the AD-group (*p* = 0.004, *p* = 0.006) and only in lesional skin of the AP-group (*p* < 0.001). Moreover, there was a significantly higher increase in loricrin observed in non-lesional skin of the AD-group than in the AP- (*p* = 0.007) and PN-groups (*p* = 0.011) (Figure 3 and Figure 5). 

**Figure 6:**
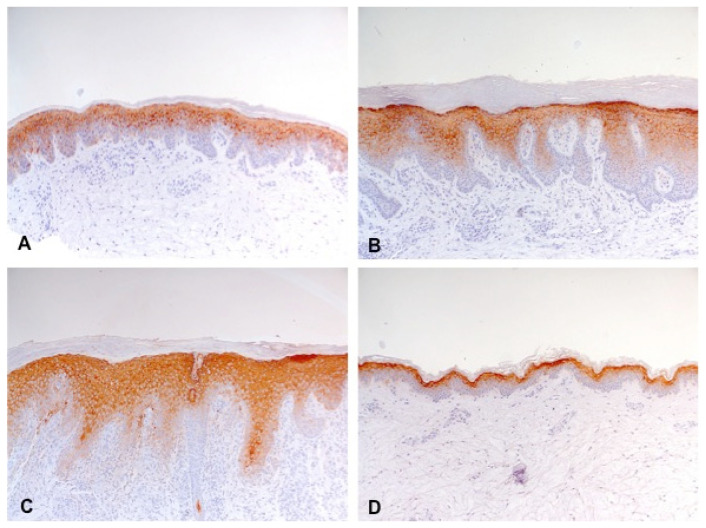
Increased expression of involucrin (extension and thickening of the brown staining line) in lesional skin of classic atopic dermatitis (**A**), atopic prurigo (**B**), and non-atopic prurigo nodularis (**C**) compared with healthy controls (**D**) shown by immunohistochemistry (100-fold magnification).

Involucrin was distinctly and significantly increased compared with the C-group in lesional and non-lesional skin of the AD-group (*p* < 0.001, *p* = 0.001) and only in lesional skin of the AP- (*p* < 0.001) and PN-groups (*p* < 0.001). Besides, a significantly higher increase in involucrin was observed in the lesional skin of the PN-group compared with the AD-group (*p* = 0.03), but not compared with the AP-group (Figure 3 and Figure 6).

### 3.5. Intercellular Lipid Lamellae

**Figure 7:**
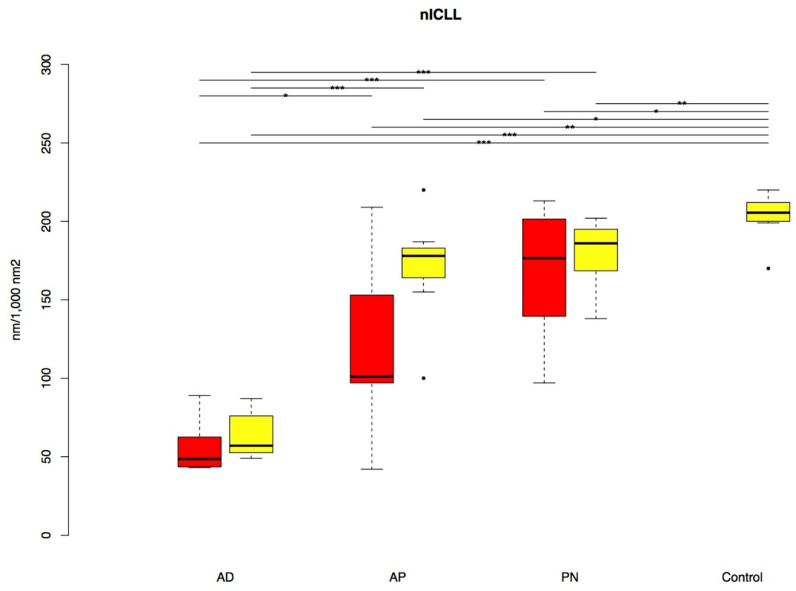
nICLL in lesional (red) and non-lesional (yellow) skin (boxplots with outliers given as single dots). *p*-value: * *p* < 0.05, ** *p* < 0.01, *** *p* < 0.001. nICLL: normalized intercellular lipid lamellae; AD: classic atopic dermatitis; AP: atopic prurigo; PN: non-atopic prurigo nodularis.

**Figure 8:**
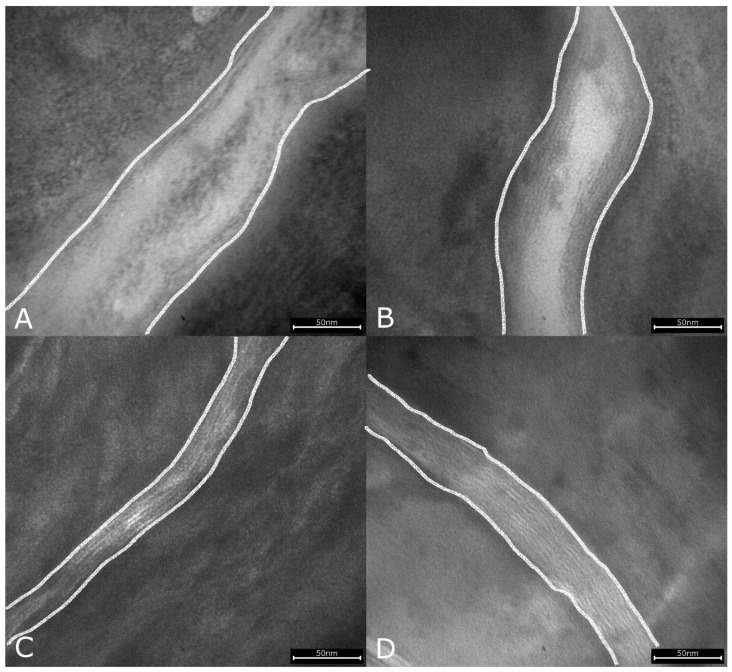
Reduced intercellular lipid lamellae (transitions from the corneocytes to the intercellular space marked with lines) in lesional skin of classic atopic dermatitis (**A**), atopic prurigo (**B**), and non-atopic prurigo nodularis (**C**) compared with healthy controls (**D**) shown by electron microscopy (245.000-fold magnification).

TEM analysis revealed significantly reduced nICLL in both lesional (*p* < 0.001, *p* = 0.004, *p* = 0.028) and non-lesional skin (*p* < 0.001, *p* = 0.03, *p* = 0.005) in all patient groups AD, AP and PN compared with the C-group. Apart from this, a significantly higher reduction in the nICLL appeared in the AD-group in both lesional and non-lesional skin compared to the AP- (*p* = 0.02, *p* < 0.001) and PN-groups (each *p* < 0.001) (Figure 7 and Figure 8).

### 3.6. FLG-Mutations

We found no homozygous carriers of the *FLG*-mutations. Heterozygous carriers were only identified for the *FLG*-mutation 2282del4; namely, 22% for the AD-, 0% for the AP-, 11% for the PN- and 10% for the C-groups. There were no significant differences between the groups, however. 

## 4. Discussion

### 4.1. Medical History Features

It is well known that PN typically manifests at higher ages, whereas the majority of patients with AD develop the disease in childhood [1,3,14]. Although not significant, there was a tendency towards AP disease onset in adulthood in our study. This fits with the assumption that special forms of AD such as AP occur particularly in the adult-onset form [1,2].

Data on allergic asthma, pollen allergy, and positive family history of atopic diseases in first-degree relatives illustrate the common atopic background of the AD and AP groups—even if there are some missing significant differences, especially of the other atopic disorders, allergic rhinitis, and further allergies. Nevertheless, the atopic groups show altogether higher frequencies of these atopic parameters than the PN group.

Although there is evidence in the literature for an association of AD with overweight or obesity [33], there is currently no evidence of elevated BMI in prurigo forms as observed in this study. However, in the case of non-atopic PN, underlying internal diseases such as diabetes mellitus type 2, hypothyroidism, choledocholithiasis may be associated with obesity [14,17,18]. Only one of the nine subjects in our PN-group reported such a metabolic disorder, but it is possible that the remaining subjects have not yet been diagnosed with such an underlying disease. Another explanatory approach for both prurigo groups having higher BMI values is the intense pruritus—with often unsuccessfully therapeutic attempts [13]—and thus reduced quality of life—including sleep disturbance, mental stress, and social isolation [14]. We also observed this in our study—even if the elevated itch intensity and the limited life quality in the prurigo groups did not reach significance compared to the AD-group. So, it is quite possible that the heavy psychosocial burden in the prurigo groups is associated with an increased food intake and thus leads to overweight [29,34,35].

A consequence for clinical practice could be treating the underlying disease as well as considering and, if possible, improving the psychosocial situation of these patients by training of relaxation techniques or cognitive behavior therapy, including education about the patient’s given prurigo disease. The effectiveness of standardized training programs similar to the educational programs for AD (so-called eczema school) has been little researched to date [1,21,23,24].

Only in the AD group was the use of daily skincare significantly more frequent than in the skin-healthy controls, which suggests that basic skincare is not well implemented in the prurigo groups.

### 4.2. Clinical Characteristics

The distinctly increased clinical scores in all three disease groups lacking significant differences illustrate the resemblance of the three disorders in terms of disease severity. However, the relatively low values for current itch intensity and for DLQI, albeit they are both entirely subjective parameters for disease severity, were surprising in our population in all three disease groups. In other studies, in contrast, itch intensity for PN and DLQI for AD were on average approximately twice as high as in our study [17,29]. The grading of our values as average mild is in accordance with the usual classification of NRS in the itch assessment [28]; for the DLQI, an established classification is missing. There was no correlation with the SCORAD, as all disease groups presented on average moderate values according to the common SCORAD classification [23]. The most likely cause for the comparatively low pruritus and the correspondingly only slight reduction in life quality of our patient collective must be assumed to be effective therapy and satisfactory care. Therapeutic measures including systemic medication were not an exclusion criterion for participation in our study. In principle, however, a wide range of tolerance levels for itch are known [23]. 

The SCORAD is a globally widely used and well-evaluated scoring system for AD [23,36]. However, it has to be critically considered regarding the possible inter- and intraobserver-variability caused by the subjective assessment [37] as well as the fact of being a snapshot variable, which thus does not reflect the overall disease activity [12]. Furthermore, the SCORAD is designed only for AD and not for other pruritus-associated dermatoses—accordingly, there has not been any use of the SCORAD for non-atopic PN in the literature as of yet. Only recently, the validated Prurigo Activity Score (PAS) was created for the assessment of disease severity of chronic prurigo [38], but its use would have evoked the question of comparability of the two scores SCORAD to PAS between the different groups, along with the problem of determining which score would have been more suitable for AP (as “intersection disease” of AD and PN). For AP, the comparable application of a similar, classic severity score of AD (i.e., Eczema Area and Severity Index [EASI]) can be found in the literature [39]. Nevertheless, it remains questionable whether the applicability of the SCORAD is simply transferable to non-atopic PN as implicated in our study.

A critical point of the DLQI, which, like the SCORAD, is a frequently used tool due to its brevity and simplicity—is its unidimensionality, which is shown by the underrepresentation of the emotional aspect of life quality. This deficit may have contributed to the low DLQI values in our study [40].

Xerosis is a known common feature of AD [3]. It was significantly increased in the AD group in contrast to the three other groups, which indicates a particularly pronounced barrier disorder in classic AD.

Meanwhile, seasonal differences should be taken into account when assessing xerosis, e.g., the per se already dry skin in winter [41,42].

### 4.3. Hydration and TEWL

The reduced SC hydration in lesional skin of all three disease groups, as well as the increased TEWL, which is the most commonly used marker for barrier disruption, demonstrate a disrupted skin barrier in all three disorders. This disturbed epidermal water balance is supported by our findings of decreased filaggrin and consequently reduced filaggrin degradation products as well as by reduced intercellular lipid lamellae, both of which contribute to water retention in the SC [4]. However, significantly reduced hydration even in non-lesional skin was only found in AD, matching the significantly increased xerosis observed in AD, also signifying a particularly severe barrier disruption in classic AD. Reduced hydration in lesional and non-lesional skin is well known for AD, as is increased TEWL in lesional skin [8]. Controversial results exist regarding increased TEWL in non-lesional skin of AD [8,43], which we did not detect significantly in any disease group. There are no comparative studies for PN regarding SC hydration and TEWL.

Some of the influencing factors in the biophysical measurements, such as temperature, humidity, and light conditions in the examination room, were largely adapted in our study [44]. However, low atmospheric humidity can lead to reduced TEWL [45], just as the topical application of dermatotherapeutics including emollients as well as glucocorticoids may fake an improved skin barrier [5]—as sometimes suspected in TEM analysis despite prior instruction of a temporary renunciation of any external agents. On the other hand, long-term use of glucocorticoids can impair the barrier function [46]. At last, it should be considered that these biophysical parameters only partially reflect the barrier function since SC hydration and TEWL only refer to the “inside-out” barrier.

### 4.4. Histological Features and Epidermal Differentiation

The severe disruption of the epidermal barrier function that was revealed by reduced hydration and increased TEWL is related to changes in epidermal proliferation and differentiation as well as inflammation. The thickened epidermis we observed in lesional skin of all three disease groups, which resulted from increased epidermal proliferation triggered by inflammation [4], have already previously been described as histological features in both AD and PN. The distinctive hyperkeratosis and hypergranulosis in lesional skin of all three disease groups are equally known for AD and PN [47,48].

The fact that in the AD-group, even the non-lesional skin showed significant hyperkeratosis compared to the other groups supports the assumption of a particularly severe barrier disruption in classic AD. This is also well compatible with reduced hydration [42,45].

The impaired epidermal differentiation was reflected in reduced filaggrin and increased loricrin and involucrin expression in all three disease groups. 

However, a significant reduction in filaggrin, even in non-lesional skin, was missing, even though it has been previously described for AD [8,49]; currently, there are no previous studies for PN regarding filaggrin. 

On the other hand, loricrin and involucrin were significantly elevated in the AD-group even in non-lesional skin, underlining the particularly pronounced barrier disorder in classic AD. 

The pathophysiological mechanisms and implications of the observed increased expression of loricrin and, in particular, involucrin in all three clinical pictures are still unclear. It might be a result from the reduced filaggrin expression in a compensatory way, similarly to the increased expression of small proline-rich proteins and repetin with a barely affected barrier previously seen in studies with *LOR*-knockout-mice; however, the absence of any CE-proteins did not automatically induce an increase in other CE-proteins [50,51,52,53]. The complexity of this system of differentiation is also shown by our results of the skin healthy controls, which displayed elevated involucrin with minimally reduced loricrin, which in turn suggests a compensatory mechanism between these two CE-proteins. For both loricrin and involucrin, the literature contains opposite findings regarding AD [8,9,49,54,55,56]. In regard to PN, there are currently no previous studies about involucrin and merely one with an indicated lack of loricrin [54]. For healthy skin, no altered expression of any CE-proteins has been described in previous studies [8,9,55]. However, Cau et al. [45] found similar changes as in our skin healthy controls under dry environmental conditions, namely an increase in involucrin and a decrease in loricrin.

Hence, it can be assumed that these two epidermal differentiation proteins may be quite variably expressible CE-components in some cases where a deficiency in the CE-construct exists. However, as seen in this study and in another, such compensatory attempts are insufficient to restore the barrier normality in these instances of complex disease because of the multiple influencing factors [56].

In principle, reduced filaggrin content can also be explained by low humidity via reduced SC hydration, resulting in increased filaggrin degradation to its metabolites [42,45] or by long-term topically applied glucocorticoids [57].

### 4.5. Intercellular Lipid Lamellae

The ICLL analysis was the only parameter that showed a notable reduction in all three disease groups both in lesional as well as in non-lesional skin, with a maximum reduction in the AD-group, illustrating once again the particularly severe barrier disruption in classic AD. The disturbed intercellular lipid architecture of the SC is well known for AD [6,9] but not yet for PN.

Reduced lipid content can also be caused by the cold season [41], short- or long-term topical treatment with glucocorticosteroids, or stress caused by enhanced endogenous glucocorticosteroid production [46,58,59]; the chosen anatomical site does not influence the measurement of the ICLL [6]. 

### 4.6. FLG-Mutations

*FLG*-mutations are strongly associated with AD [10,11,12]. Here, we did not observe significant differences between the disease groups with regard to the frequency of *FLG* mutations. However, the small number of *FLG* mutation carriers in the AD-group, as well as their occurrence in the C-group, reflect the fact that many patients with AD do not have an *FLG*-mutation at all, while likewise, healthy people can well carry single *FLG*-mutations without developing AD [60]. The percentages of *FLG*-mutation in the AD- and the C-groups approximately correspond with the ones observed in previous studies for AD and healthy individuals [5,61]. The absence of homozygous carriers is in accordance with a former study as well [11]. However, the detection of the other common *FLG* mutations, especially of R501X, was missing in our study population. Currently, no investigations exist on the carrier state of the *FLG*-mutation in PN. The fact that patients from all three disease groups showed a filaggrin deficiency in lesional skin even in the absence of *FLG*-mutations confirms the impact of other factors, which can cause a reduction in filaggrin expression, e.g., cutaneous cytokine imbalance in the context of Th2 predominance or mechanical barrier damage from the scratching itself (so-called itch-scratch cycle) [22,60].

## 5. Conclusions

Our study showed that classic AD, AP, and non-atopic PN resemble each other closely in their disease severity as well as their impaired epidermal barrier structure and resulting disturbed barrier function. The most striking feature of all three disorders was the reduced lipid lamellae of the SC, representing an epidermal barrier defect as well as disturbed epidermal differentiation as a sign of skin barrier disruption. 

A decidedly impaired epidermal barrier in PN has now actually been proven, leading to the recommendation of consistent and intensive skincare not only for patients with AD but also for those with non-atopic PN to improve skin lesions and reduce itch. Although basic therapy is recommended for non-atopic PN according to the guidelines, it is not a regular part of the therapy concept or rather not firmly established in the therapeutic awareness of the patients—a fact that was also evident in our study population. The expected beneficial effect of basic therapy on skin barrier repair in PN would need to be confirmed by prospective studies. 

Classic AD stood out among the three diseases since the skin barrier impairment was partly detected to a significantly greater extent even in non-lesional skin, and the clinical correlate, namely xerosis, was present more clearly as well. These findings point toward a barrier impairment comprising the whole skin integument in classic AD, which not only confirms the current state of scientific research but also further supports the therapeutic guidelines and recommendations established to date for AD. In contrast, significant barrier impairment was largely confined to lesional skin in the prurigo groups. 

Several interesting results, including increased BMI in the prurigo groups and the enhanced expression of the epidermal differentiation proteins loricrin and involucrin in all patient groups, could serve as the basis for future research, as the causes and implications of which are yet unclear. 

A major limitation of this study is the small sample size resulting in low statistical power, which could explain some missing statistical significance. Further studies with a larger patient collective are necessary to confirm our results. 

In summary, all three diseases, AD, AP, and PN, share the feature of a disturbed epidermal barrier. We, therefore, suggest that regular barrier-enhancing skincare should also be an integral part of management strategies for non-atopic prurigo nodularis.

## Figures and Tables

**Table 1:** Clinical characteristics and epidermal barrier parameters of the four study groups.

Relative (Absolute) Frequency, Median [1., 3. Quartile] or Mean ± 1 SD	AD (*n* = 9)	AP (*n* = 7)	PN (*n* = 9)	Control (*n* = 10)	*p* *
Age at disease onset (years)	11 [5.5, 21]	28 [6, 40]	43 [30, 49.5]	-	0.016
Pollen allergy (%)	78 (7)	14 (1)	0 (0)	0 (0)	<0.001
Allergic asthma (%)	33 (3)	71 (5)	0 (0)	30 (3)	0.025
Positive family history for atopic diseases (%)	78 (7)	86 (6)	22 (2)	50 (5)	0.035
BMI (kg/m^2^)	22.4 [20.2, 26]	28.6 [27.1, 34.9]	30.2 [26.7, 49.9]	23.5 [20, 25.2]	<0.0001
Daily skin care (%)	56 (5)	43 (3)	33 (3)	0 (0)	0.875
SCORAD (points)	39 ± 12.5	32.4 ± 17.6	37.3 ± 5.7	0.3 ± 0.9	0.0001
Itch intensity (points)	2.5 ± 2.5	3.1 ± 2.7	3.7 ± 3.2	0.2 ± 0.4	0.018
DLQI (points)	7 ± 4.1	9.1 ± 8.6	10.6 ± 7.8	0.1 ± 0.3	0.003
Xerosis (points)	1.6 ± 1.1	0.3 ± 0.5	0.4 ± 0.7	0.1 ± 0.3	0.001
Hydration L (“arbitrary units”)	18.7 [15.7, 27.4]	27.3 [20, 40.3]	17 [8.6, 20.8]	-	0.13
Hydration NL (“arbitrary units”)	26.3 [22.3, 3.4]	37 [24, 46.7]	28.7 [21.2, 38.2]	44.9 [33.2, 51.1]	0.085
TEWL L (g/hm^2^)	22.1 [17.2, 37.7]	18.6 [10.8, 44.4]	14.7 [11.1, 24]	-	0.122
TEWL NL (g/hm^2^)	7.3 [6.7, 13.6]	7.1 [6.8, 13]	6.6 [4.8, 7.2]	6.7 [6, 9.7]	0.403
Epidermal thickness L (points)	2.7 ± 0.4	2.3 ± 1.1	2.5 ± 0.6	-	0.474
Epidermal thickness NL (points)	0.3 ± 0.6	0 ± 0	0 ± 0	0.1 ± 0.2	0.109
Inflammation L (points)	2.2 ± 0.7	2.2 ± 1.1	2.4 ± 0.7	-	0.849
Inflammation NL (points)	0.2 ± 0.4	0 ± 0	0 ± 0	0 ± 0	0.057
Hyperkeratosis L (points)	2.6 ± 0.5	2.5 ± 1.2	2.5 ± 0.7	-	<0.001
Hyperkeratosis NL (points)	1.0 ± 0.5	0 ± 0	0 ± 0	0.4 ± 0.5	<0.001
Hypergranulosis L (points)	1.9 ± 0.8	1.9 ± 1.1	1.6 ± 1.3	-	0.001
Hypergranulosis NL (points)	0.3 ± 0.5	0 ± 0	0 ± 0	0.2 ± 0.4	0.184
Filaggrin L (points)	−0.6 ± 0.5	−0.5 ± 0.4	−0.7 ± 0.4	-	0.735
Filaggrin NL (points)	−0.2 ± 0.4	0 ± 0	−0.1 ± 0.2	0 ± 0	0.329
Loricrin L (points)	0.8 ± 0.7	1.7 ± 0.8	1.1 ± 1.2	-	0.257
Loricrin NL (points)	0.6 ± 0.4	0 ± 0	0.1 ± 0.2	−0.1 ± 0.3	<0.001
Involucrin L (points)	2.4 ± 0.5	2.5 ± 0.4	2.9 ± 0.2	-	0.06
Involucrin NL (points)	1.6 ± 0.3	1.1 ± 0.9	0.9 ± 0.8	0.4 ± 0.5	0.005
nICLL L (nm/1.000 nm^2^)	48.3 [43.5, 62.5]	101 [97, 153]	176 [139, 201.5]	-	<0.001
nICLL NL (nm/1.000 nm^2^)	57 [52.5, 76]	178 [164, 183]	186 [168.5, 195]	205 [200, 212]	<0.001

* Overall *p* values from χ^2^ test (pollen allergy, allergic asthma, positive family history for atopic diseases, daily skincare) or one-way ANOVA for normally distributed variables (SCORAD, Itch intensity, DLQI, Xerosis, Epidermal thickness L and NL, Inflammation L and NL, Hyperkeratosis L and NL, Hypergranulosis L and NL, Filaggrin L and NL, Loricrin L and NL, Involucrin L and NL) and the Kruskal-Wallis test for non-normally distributed variables (Age at disease onset, BMI, Hydration L and NL, TEWL L and NL, nICLL L and NL); AD: classic atopic dermatitis; AP: atopic prurigo; PN: non-atopic prurigo nodularis; L: lesional; NL: non-lesional; SCORAD: severity scoring of atopic dermatitis; DLQI: dermatology life quality index; TEWL: transepidermal water loss; nICLL: normalized intercellular lipid lamellae.

## Data Availability

The Data presented in this study are available on request from the corresponding author. The data are not publicly available due to ethical prescriptions.
